# Thrombolysis for acute ischemic stroke by tenecteplase in the emergency department of a Moroccan hospital

**DOI:** 10.11604/pamj.2015.21.37.6491

**Published:** 2015-05-19

**Authors:** Ahmed Belkouch, Said Jidane, Naoufal Chouaib, Anass Elbouti, Tahir Nebhani, Rachid Sirbou, Hicham Bakkali, Lahcen Belyamani

**Affiliations:** 1 Emergency Department, Mohamed V Military Hospital of Instruction, Faculty of Medicine and Pharmacy, Rabat, Morocco

**Keywords:** Acute ischemic stroke, thrombolysis, tenecteplase

## Abstract

**Introduction:**

Thrombolysis has radically changed the prognosis of acute ischemic stroke. Tenecteplase is a modified form of rt-PA with greater specificity for fibrin and a longer half-life. We report the experience of a Moroccan tertiary hospital in thrombolysis using Tenecteplase.

**Methods:**

We conducted an open prospective study of all patients who were treated with Tenecteplase for an acute ischemic stroke admitted to our emergency department. Tenecteplase was administered intravenously at a dose of 0.4 mg/kg single bolus. The primary outcome measure was the proportion of patients achieving significant early neurological recovery defined as an improvement of 4 or more points on the NIHSS score at 24h.

**Results:**

13 patients had been treated by intravenous thrombolysis. 31% were women. Mean age was 63 years old. The mean NIHSS score at admission was 14.3 and 24h after was at 9.1. The right middle cerebral artery was involved in 69% of cases. The carotid atherosclerosis was predominant 63.3% and the cardio embolic etiology 27%. The mean time to the first medical contact after the onset of symptoms was 3h 30 min. One patient presented a capsulo-lenticular hematoma of 5 mm^3^ in the same side of the ischemic stroke.

**Conclusion:**

Tenecteplase is a more interesting thrombolytic than alteplase, it seems to be more suitable for thrombolysis in our center.

## Introduction

The intravenous thrombolysis with rt-PA has radically changed the prognosis of stroke [1–3]. Scholarly societies recommend the admission of these patients and the realization of thrombolysis in stroke units [4, 5]. The establishment of such units in developing countries, such as Morocco, encounters difficultiesand these units are slow to start. Other barriers are encountered, as short therapeutic window, the lack of information of the population that continues to ignore the main clinical signs and the absence of pre-hospital care standardized. This shows the complexity of this problem in our country. Classically, Alteplase is used to perform this type of thrombolysis; however, some studies have shown the superiority of Tenecteplase [6–8], its ease of handling and the time of its action to better take advantage of the short window therapy. Our choice was instinctively focused on thrombolysis by Tenecteplase that seemed more suited to our context. We report our experience in thrombolysis for acute ischemic stroke in 13 patients all treated with Tenecteplase (Metalyse^®^), they were all registred at the emergency department of the Mohammed V Military Hospital of instruction, which is a tertiary hospital in Rabat. Our emergency department has within it an emergency intensive care unit. To our knowledge no study of this kind has been made in Morocco.

## Methods

We conducted an open prospective study of all patients who were treated with Tenecteplase for anacute ischemic stroke admitted to the emergency department of the Military Hospital Mohammed V. the whole departments taff received a training about thrombolysis with awareness to the importance of time factor “time is brain”. Physicians have been also trained to the assessment of neurological status by calculating the NIHSS score. This score counts 15 items that measure the depth of the neurological deficit quoted from 0 to 42, <5 it reflects a minor deficit and > 25 severe neurological impairment. In consultation with the medical imaging department, access to MRI was facilitated during the day and to the CT scan during the night. The presence of a neurologist was requested without delaying patients care. All patients who were admitted to our department in the period between June 2010 and October 2014, with symptoms suggestive of Stroke 4h30min after onset were immediately assessed by a senior emergency physician to decide on their Eligibility for intravenous thrombolysis. We first conducted a quick but detailed clinical examination with capillary blood glucose monitoring, record of personal and family medical history, calculation of NIHSS score. At the same time we did standard laboratory tests, ECG and chest X-ray. We have respected the same inclusion and exclusion criteria as those adopted in the ECASS 3 study [3] (Table 1). The family consent, after discussion of the benefit and complications of this treatment was obtained for each patient who could not give it himself. Tenecteplase was administered intravenously at a dose of 0.4 mg/kg single bolus. Patients who received thrombolytic therapy were the subject of a prospective study. The collected variables were the time of onset of symptoms, the time of arrival to the emergency room, demographic data, risk factors for ischemic stroke, a detailed neurological examination with calculation of the NIHSS score (which was done on admission, 1h, 2h, and 24h after administration of Tenecteplase), monitoring of systolic and diastolic blood pressure and blood glucose on admission, time of completion of the imaging and its results, Tenecteplase administration time. Types of stroke were defined by the Criteria used in TOAST study [9]. The primary outcome measure was the proportion of patients achieving significant early neurological recovery defined as an improvement of 4 or more points on the NIHSS score at 24h. The safety endpoint was the rate of symptomatic intracranial hemorrhage (SICH) and death. SICH was defined according to the NINDS rt-PA/Cochrane criteria as any new evidence of intracranial blood on CT or magnetic resonance imaging (MRI) accompanied by a neurological deterioration of 4 or more points on the NIHSS score from baseline.

**Table 1:** Exclusion and inclusion criteria according to the NINDS and ECASS 3 studies and which were adopted in our study

Main inclusion criteria	Main exclusion criteria
Acute ischemic stroke	Intracrânial haemorrhage
Age, 18 to 80 years	Time of symptom onset unknown
Onset of stroke symptoms 3 to 4.5 hours before initiation of study-drug administration	Symptoms rapidly improving or only minor before start of infusion
Stroke symptoms present for at least 30 minutes with no significant improvement before treatment	Severe stroke as assessed clinically (e.g., NIHSS score >25) or by appropriate imaging techniques*
	Seizure at the onset of stroke
	Stroke or serious head trauma within the previous 3 months
	Combination of previous stroke and diabetes mellitus
	Administration of heparin within the 48 hours preceding the onset of stroke, with an activated partial-thromboplastin
	Time at presentation exceeding the upper limit of the normal range
	Platelet count of less than 100,000 per cubic millimetre
	Systolic pressure greater than 185 mmHg or diastolic pressure greater than 110 mmHg, or aggressive treatment (intravenous medication) necessary to reduce blood pressure to these limits
	Blood glucose less than 50 mg per decilitre or greater than 400 mg per decilitre
	Symptoms suggestive of subarachnoid haemorrhage, even if imagery was normal
	Oral anticoagulant treatment
	Major surgery or severe trauma within the previous 3 months
	Other major disorders associated with an increased risk of bleeding

* A severe stroke as assessed by imaging was defined as a stroke involving more than one third of the middle cerebral artery territory. NIHSS denotes National Institutes of Health Stroke Scale in which total scores range from 0 to 42, with higher values reflecting more severe cerebral infarcts

## Results

From June 2010 to October 2014, 13 patients had undergone intravenous thrombolysis using Tenecteplase at maximum of 4h30min after the onset of a neurological impairment following an acute ischemic stroke in the carotid territory. 31% were women (4/13). The mean age was 63 years (± 15). Most of them arrived to the hospital using their own vehicle (10/13 or 77%), or after a call to the emergency department from the family doctor followed by a non-medical transport to hospital (23%). The mean NIHSS score at admission was 14.3 (±7), 2 hours after thrombolysis it was 12.8 (±8) and 24h after was at 9.1 (± 9). We also noted that 77% of our patients have improved their score NHISS more than 4 points. Prior to injection of thrombolytic, brain CT scan without contrast was performed in 5 patients (38%) showing early signs of ischemia (dedifferentiation cortico-subcortical, deletion of the insular ribbon, deletion of the lenticular nucleus, spontaneous and asymmetric hyper density of the middle cerebral artery). Brain MRI diffusion / perfusion was performed for the remaining patients (8/13 or 62%). We respected the deadline for the treatment of 4h30min after the onset of symptoms for all our patients. Strokes in the territory of the right middle cerebral artery represented 69% (9/13) of cases. The carotid atherosclerosis was predominant (63.3%) and the cardioembolic etiology (27%). Undetermined causes accounted for the rest of cases (9.7%) (Table 2). The mean time to the first medical contact after the onset of symptoms was 3h 30 min (±35min). The average “door to treatment” time, or the time between hospital admission and the beginning of the injection of fibrinolytic treatment was 40 minutes (±20min). Only one patient presented a capsulo lenticular hematoma of 5 mm^3^ in the same side of the ischemic stroke. He presented an uncontrolled high blood pressure 220/130 mmHg, half an hour after administration of thrombolytic therapy. Control CT scans, showed that the hematoma had kept the same volume without requiring surgery. No deaths orother complications were noted.

**Table 2:** Baseline characteristics of our 13 patients on admission compared with the NINDS study

Baseline characteristics study	Our study	NINDS
Age (mean ± S.D)	63±15	68±11
Female (%)	31	42
Weight (kg)	60±11	76±16
NIHSS score median (range)	14.3 (±7)	14 (1-37)
Initial plasma glucose (mg/dl)	134±48	149±71
Systolic blood pressure (mmHg)	160±18	155±22
Diastolic blood pressure (mmHg)	80 ±17	85±13
Stroke subtype (%)		
Cardioembolism	27	43
Large vessel occlusive	41.3	37
Small vessel occlusive	22	16

## Discussion

All over the world, one person every 5 seconds undergoes a stroke. Labbé O. & Massart J. (2010) [10]. Moreover, in France, the annual incidence of stroke is 1.6 to 2.4 per 1000 people, representing from 100000 to 145000 strokes per year, 15 to 20% of them will die after the first month and 75% them will survive with sequelae. In Morocco, the results of the survey of stroke, conducted among 13,000 households between November 2008 and May 2009, in the region of Rabat-Salé-Zemmour-Zaer and Grand Casablanca, revealed 90,000 cases of which 50,000 came from the rural against 40,000 from the urban areas. The working group ECASS3 had set a target to treat patients within four and a half hours after the onset of symptoms, with an intra-hospital delay “door to treatment” of up to 1 hour. In our series all patients were treated respecting this delay of 4h30min after symptom onset. This reinforces the need to educate health professionals and the general public about the stroke symptoms, so that these patients can be quickly identified and treated. The promotion of pre-hospital medicine is the key to improving these deadlines. In our series the intra hospital management delay was less than one hour (mean time: 40 minutes), thanks to the involvement of all stakeholders. In the other series, this time ranges from 96 minutes [10] to 33 min [11]. It should be reduced by convening more meetings of the various stakeholders and raising awareness of the fact that faster is the treatment better are the results. Tenecteplase is a modified form of rt-PA with greater specificity for fibrin and a longer half-life. Our study confirms the benefits seen in other studies comparing Tenecteplase with Alteplase and have shown that the clinical benefit is not burdened with increased bleeding [6–8]. Indeed, at a dose of 0.4 mg/kg we have encountered a single bleeding episode which was due to an uncontrolledhigh blood pressure and has not worsened after blood pressure control. After thrombolysis significantly reduction of the NIHSS score was observed in 77% of our patients. NHISS average score on admission was 14.3 and it has fallen to 9.1 after 24 hours. Major neurological improvement, defined by Saposnik & al [12] by a NHISS score of 0 points or an improvement of more than 8 points, was noted in 7 of our patients (54%) (Table 3) [1, 13–15]. Our results are probably due to the rigorous selection of patients eligible for thrombolysis and respect for different time frames.

**Table 3:** Proportions of patients who have shown an improvement of more than 4 points on the NHISS score in several studies

Different studies	Number of patients	Percentage of amelioration of NIHSS score%
HMIMV	13	77,0
New delhi (13)	54	66,7
GSH (14)	42	67,0
Thailand 2006 (15)	34	70,6
NINDS rt-PA (1)	312	47,0

## Conclusion

The protocol of thrombolysis, used in our center to date, seems convincing. Tenecteplase is a more interesting thrombolytic than Alteplase, which has proven itself in the thrombolysis in myocardial infarction. It seems to be more suitable for thrombolysis in centers that are not equipped with stroke units as well as for countries where pre-hospital medicine is in its beginnings. The short time of its action allows to better taking advantage of the short window therapy. Confirmation of its benefits requires studies on larger cohorts.

## References

[1] (1995). Tissue plasminogen activator for acute ischemic stroke. The National Institute of Neurological Disorders and Stroke rt-PA Stroke Study Group. N Engl J Med..

[2] Hacke W, Kaste M, Fieschi C, Toni D, Lesaffre E, von Kummer R (1995). Intravenous thrombolysis with recombinant tissue plasminogen activator for acute hemispheric stroke. The European Cooperative Acute Stroke Study (ECASS) JAMA..

[3] Hacke W, Kaste M, Bluhmki E, Brozman M, Dávalos A, Guidetti D (2008). Thrombolysis with alteplase 3 to 45 hours after acute ischemic stroke. N Engl J Med..

[4] Stroke∣ introduction∣ Guidance and guidelines∣ NICE (Internet). https://www.nice.orguk/guidance/cg68/chapter/introduction.

[5] AVC prise en charge précoce - Recommandations [Internet]. http://www.hassante.fr/portail/plugins/ModuleXitiKLEE/types/FileDocument/doXitijsp?id=c_830201.

[6] Haley EC, Lyden PD, Johnston KC, Hemmen TM, TNK in Stroke Investigators (2005). A pilot dose-escalation safety study of tenecteplase in acute ischemic stroke. Stroke.

[7] Parsons M, Spratt N, Bivard A, Campbell B, Chung K, Miteff F (2012). A Randomized trial of tenecteplase versus alteplase for acute ischemic stroke. New England Journal of Medicine..

[8] Logallo N, Kvistad CE, Nacu A, Naess H, Waje-Andreassen U, Asmuss J (2014). The norwegian tenecteplase stroke trial (NOR-TEST): randomised controlled trial of tenecteplase vs alteplase in acute ischaemic stroke. BMC Neurology..

[9] Adams HP, Bendixen BH, Kappelle LJ, Biller J, Love BB, Gordon DL (1993). Classification of subtype of acute ischemic stroke Definitions for use in a multicenter clinical trial TOAST Trial of Org 10172 in Acute Stroke Treatment. Stroke..

[10] Wang DZ, Rose JA, Honings DS, Garwacki DJ, Milbrandt JC (2000). Treating acute stroke patients with intravenous tPA: the OSF stroke network experience. Stroke..

[11] Albers GW, Bates VE, Clark WM, Bell R, Verro P, Hamilton SA (2000). Intravenous tissue-type plasminogen activator for treatment of acute stroke: the standard treatment with alteplase to reverse stroke (STARS) study. JAMA..

[12] Saposnik G, Di Legge S, Webster F, Hachinski V (2005). Predictors of major neurologic improvement after thrombolysis in acute stroke. Neurology..

[13] Padma MV, Singh MB, Bhatia R, Srivastava A, Tripathi M, Shukla G (2007). Hyperacute thrombolysis with IV rt-PA of acute ischemic stroke: efficacy and safety profile of 54 patients at a tertiary referral center in a developing country. Neurol India..

[14] Wasserman S, Bryer A (2012). Early outcomes of thrombolysis for acute ischaemic stroke in a South African tertiary care centre. S Afr Med J..

[15] Suwanwela NC, Phanthumchinda K, Likitjaroen Y (2006). Thrombolytic therapy in acute ischemic stroke in Asia: the first prospective evaluation. Clin Neurol Neurosurg..

